# Ocular manifestations of ectodermal dysplasia

**DOI:** 10.1186/s13023-021-01824-2

**Published:** 2021-05-01

**Authors:** Daphna Landau Prat, William R. Katowitz, Alanna Strong, James A. Katowitz

**Affiliations:** 1 Division of Ophthalmology, The Children’s Hospital of Philadelphia, 34Th and Civic Center Boulevard, Philadelphia, PA 19104 USA; 2 Sackler Faculty of Medicine, Tel Aviv University, Tel Aviv, Israel; 3 Division of Human Genetics, The Children’s Hospital of Philadelphia, Philadelphia, PA USA

**Keywords:** Ectodermal dysplasia, Ptosis, Lash ptosis, *EDA1*, *TP63*, Ectrodactyly-ectodermal dysplasia-clefting, EEC, Ankyloblepharon-ectodermal defects-cleft lip/palate, AEC

## Abstract

**Purpose:**

The ectodermal dysplasias (EDs) constitute a group of disorders characterized by abnormalities in two or more ectodermal derivatives, including skin, hair, teeth, and sweat glands. The purpose of the current study was to evaluate ocular manifestations in pediatric patients with ED.

**Methods:**

Retrospective case series including consecutive ED subjects who were treated in the ophthalmology department at the Children’s Hospital of Philadelphia over a 12-year period (2009–2020). Main Outcome Measures were ocular and ocular adnexal abnormalities.

**Results:**

Thirty subjects were included: 20 males (67%), mean age of 4.5 years (range 0.3–18). Patients with different subtypes were included, with the hypohidrotic ED and ectrodactyly-ectodermal dysplasia-clefting variants being most prevalent. Most common findings were: lacrimal drainage obstruction in 12 (40%) including punctal agenesis in 10 (33%), refractive errors in 13 (43%) and amblyopia in 6 (20%). A new finding of eyelid ptosis or eyelash ptosis was demonstrated in 11 subjects (37%), mostly associated with *TP63* or *EDA1* genes variants.

**Conclusion:**

Ectodermal dysplasias are associated with various ocular pathologies and amblyopia in the pediatric population. We report a possible genetic association between lash ptosis and EDA1 gene, and eyelid ptosis and TP63 or EDA1 genes variants.

## Introduction

Ectodermal dysplasias (ED) are genetic conditions affecting the development and/or homeostasis of two or more ectodermal derivatives, including skin, hair, teeth, nails, and sweat glands [1]. They constitute a large and diverse group of over 200 disorders, heterogeneous in their genetic causes and clinical phenotypes, with a variable range of reported prevalence [1–6]. The anomalies affecting the epidermis and epidermal appendages are extremely variable; many are associated with malformations in other organs and systems, thus management usually requires a multidisciplinary approach [4].

Several EDs were reported to have ocular abnormalities; however, few large cohort studies focus on ocular manifestations of these conditions [1, 4–13]. The purpose of the current study was to describe the ocular phenotype including new findings in a large cohort of pediatric subjects with various ED disorders.

## Methods

Retrospective analysis of all consecutive ED subjects who were treated at the ophthalmology department in the Children’s Hospital of Philadelphia in a 12-year period (2009–2020) was performed. Data collected included demographics, clinical photographs, symptoms, diagnosis, genetics, and management. Statistical analysis was carried out using Microsoft Excel (Microsoft, Redmond, WA, USA). The described research adhered to the tenets of the Declaration of Helsinki. The study and data accumulation were carried out with approval from the Institutional Review Board (IRB).

## Results

Thirty subjects were studied: 20 male (67%) and 10 females (33%), with a mean age of 4.5 years at first visit (range 0.3 to 18 years), and a mean follow up time of 4.0 years (range 0–17.8). Diagnoses were as follows: hypohidrotic ED in 8 patients (HED) (27% of all patients), hidrotic ED (n = 2/30, 7%), ectrodactyly-ectodermal dysplasia-clefting (EEC) (n = 8/30, 27%), ankyloblepharon-ectodermal defects-cleft lip/palate (AEC) (n = 3/30, 10%), Rapp-Hodgkin ED (n = 2/30, 7%), Marshall syndrome (n = 2/30, 7%), unspecified ED (n = 2/30, 7%), ED with immunodeficiency (n = 1/30, 3%), tricho-dento-osseous syndrome (n = 1/30, 3%), and oculo-ectodermal syndrome (n = 1/30, 3%).

The most common presenting symptoms were tearing (14 subjects, 47%), and photophobia (6 subjects, 20%). Visual acuity was appropriate for age in each eye for 24 children (n = 24/30, 80%). Thirteen subjects (n = 13/30, 43%) had refractive errors requiring spectacles. Six children (n = 6/30, 20%) had amblyopia with a visual acuity in the range of 20/25-20/600. Among those children, 4 (n = 4/30, 13%) had strabismus.

**Fig. 1:**
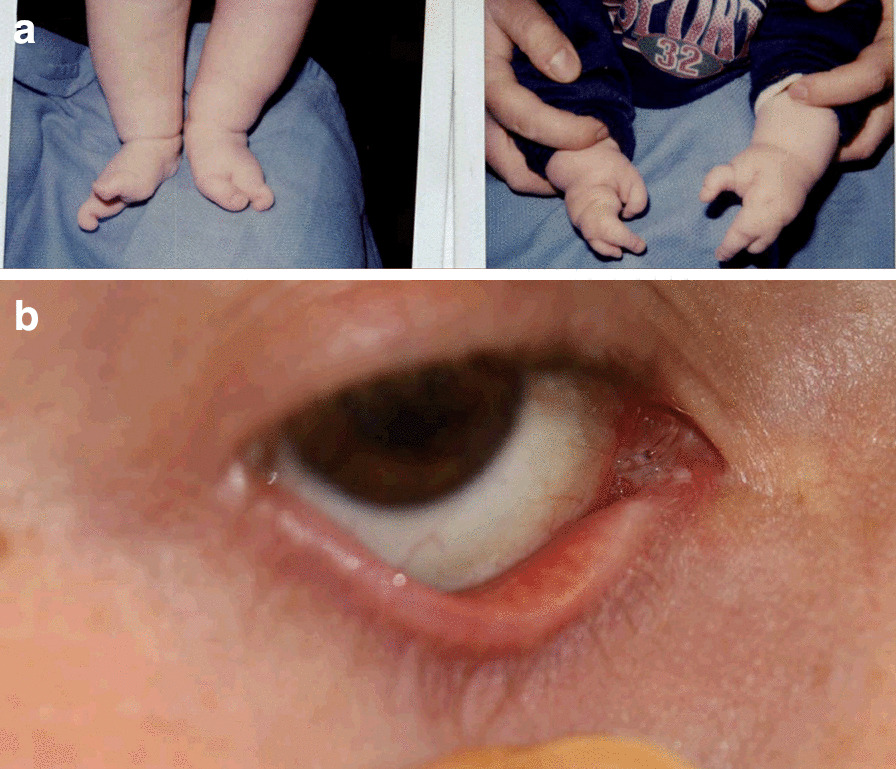
A male patient with Ectrodactyly-ectodermal dysplasia-clefting (EEC) syndrome. He had constant epiphora due to right sided punctal agenesis that resolved with CJDCR at the age of 18 years. Systemic disorders included cleft lip and palate, syndactyly, dental abnormalities, midface hypoplasia, and hearing disorders. **a** The patient at infancy, showing ectrodactyly. **b** Same patient, at age 18 years, showing absent lower punctum on the right side (punctal agenesis), and a Lester-Jones tube

**Fig. 2:**
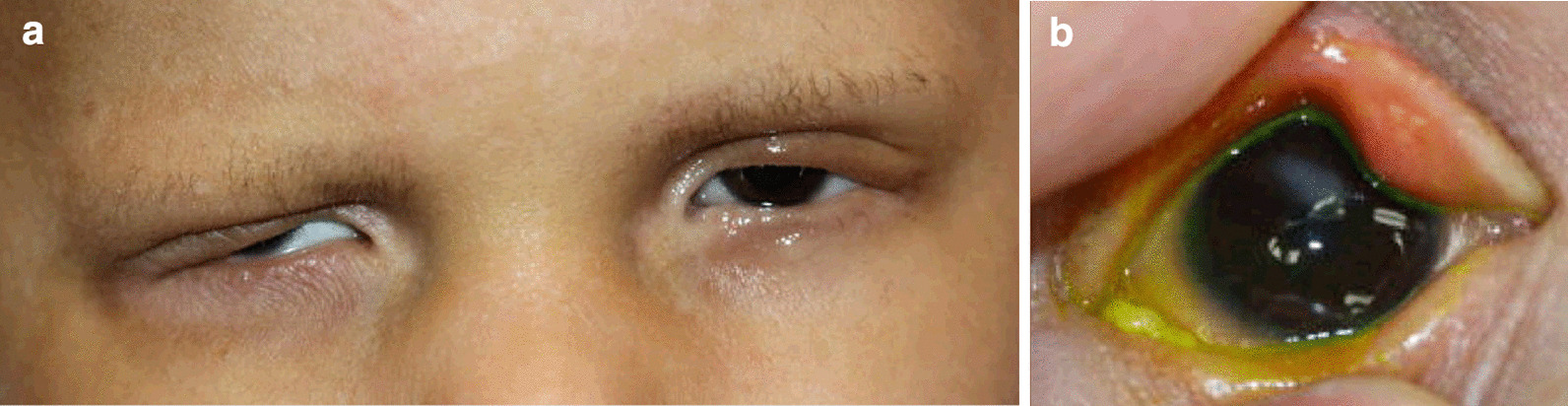
A 13-year-old male patient with Ankyloblepharon-ectodermal defects-cleft lip/palate (AEC) syndrome. The subject was extremely photophobic due to distichiasis and trichiasis (**a**) causing recurrent corneal erosions and subsequent right eye corneal scarring (**b**)

**Fig. 3:**
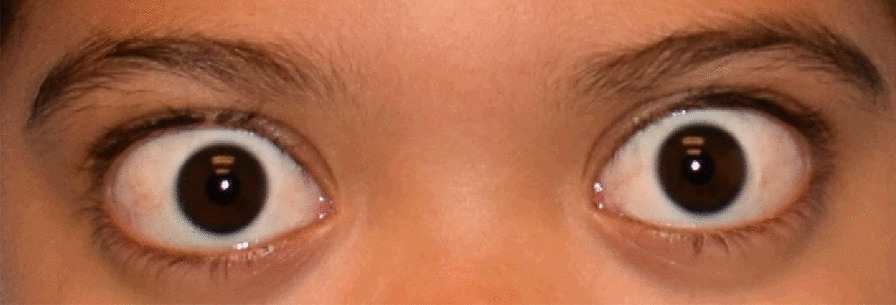
A 10-year-old male with Marshall syndrome, with high myopia (− 20D), shallow orbits and hypertelorism. The patient developed bilateral posterior subcapsular cataracts, had PPV lensectomies with prophylactic peripheral retinal laser ablation at the age of 4 years, and was left aphakic. Final visual acuity at age 10 was 20/60 OD, 20/80 OS

The most common finding was lacrimal drainage obstruction (n = 12/30, 40%), and punctal agenesis was found in 10 of these 12 subjects (n = 10/30, 33%); these were most prevalent in EEC cases (Fig. 1). Additional findings were: dry eye (n = 4/30, 13%), blepharitis (n = 3/30, 10%), allergic conjunctivitis (n = 3/30, 10%), cataracts (n = 2/30, 7%), distichiasis with trichiasis and recurrent corneal abrasions (n = 2/30, 7%) (Fig. 2). Posterior embryotoxon, posterior pole osteomas, and bilateral peripapillary colobomas were observed in a patient with oculo-ectodermal syndrome. A patient with Marshall syndrome had shallow orbits and hypertelorism on clinical impression and increased axial length, giving the appearance of lid retraction and proptosis (Fig. 3).

Twelve subjects (40%) required surgical intervention. Lacrimal drainage surgery was performed in 7 (23%) subjects, including dacryocystorhinostomy (DCR) in 4 (13%) or conjunctivodacryocystorhinostomy (CJDCR) with Jones tube placement in 3 (10%). Eyelid surgery was performed in 6 children (20%). This involved the repair of trichiasis (2), ankyloblepharon (2), ptosis (1), and lesion biopsies (1). Bilateral cataract extraction was performed in one child with Marshall syndrome. Demographics of study population, clinical presentations, and surgical interventions are summarized in Table 1.

**Table 1:** Demographics, clinical presentations, and surgical interventions of 30 pediatric subjects with Ectodermal Dysplasia

	All subtypes (total)	Unspecified ED, hidrotic and hypohidrotic	EEC	AEC	Rapp-Hodgkin ED	Marshall’s syndrome	HED with immunodeficiency	Tricho-dento-osseous syndrome	Oculo-ectodermal syndrome
Number of subjects	**30**	12	8	3	2	2	1	1	1
Gender (M:F)	**20:10**	8:4	6:2	3:0	0:2	2:0	1:0	0:1	0:1
Age at first visit, years	**4.5**	5.4	3	4.4	0.5	2.7, 0.4	3	9.5	12.5
Genetic variant (n of tests)		*EDA1 (6)*	*TP63 (3)*	*TP63 (2)*	*TP63 (2)*	*COL11-A1 (1)*	*NEMO (1)*	*DLX3 & COL1A1 (1)*	*KRAS (1)*
**Symptoms (n of subjects, %)**									
Tearing/discharge	**14, 47%**	4, 33%	7, 87%	1, 33	1	1	–	–	–
Photophobia	**6, 20%**	2, 17%	1, 14%	2, 67%	1	–	–	–	–
**Diagnoses (n of subjects, %)**									
Lacrimal disorders	**12, 40%**	2, 17%	7, 87%	2, 67%	1	–	–	–	–
Punctal agenesis	**10, 33%**	2, 17%	6, 62%	1, 33%	1	–	–	–	–
Dry eye	**4, 13%**	1, 8%	1, 14%	1, 33%	1	–	–	–	–
Strabismus	**4, 13%**	1, 8%	–	1, 33%	–	–	–	–	–
Blepharitis	**3, 10%**	1, 8%	2, 29%	–	–	–	–	–	–
With MGD	**2, 6.7%**	1, 8%	1, 14%	–	–	–	–	–	–
Allergic conjunctivitis	**3, 10%**	2, 17%	–	1, 33%	–	–	–	–	–
Distichiasis and trichiasis	**2, 6.7%**	–	–	2, 67%	–	–	–	–	–
Cataract	**2, 6.7%**	1, 8%	–	–	–	1	–	–	–
Ptosis/lash ptosis	**11,37%**	5, 42%	3, 43%	2, 67%	–	–	–	–	1
Isolated lash ptosis	**3, 10%**	2, 17%	1, 14%	–	–	–	–	–	–
Isolated ptosis	**4, 13%**	1, 8%	–	2, 67%	–	–	–	–	1
Combined ptosis & lash ptosis	**4, 13%**	2, 17%	2, 29%	–	–	–	–	–	–
Spectacles	**13, 43%**	5, 42%	3, 43%	2, 67%	–	1	–	1	1
Amblyopia	**6, 20%**	2, 17%	1, 14%	1, 33%	–	1	–	–	1
Other	**1, 3%**	–	–	–	–	–	–	–	1^a^
**Surgical intervention (n of subjects, %)**	**12, 40%**	**1, 8%**	**6, 75%**	**3, 100%**	**1**	**1**	–	–	**1**
Lacrimal repairs	**7, 23%**	–	5, 62%	1, 33%	1	–	–	–	–
Trichiasis repair	**2, 6.7%**	–	–	2, 67%	–	–	–	–	–
Ankyloblepharon repair	**2, 6.7%**	–	–	2, 67%	–	–	–	–	–
Ptosis repair	**1, 3%**	–	–	–	–	–	–	–	1
Cataract extraction	**1, 3%**	–	–	–	–	1	–	–	-
Other	**2, 3%**	1, 8%^b^	-	-	-	1	-	-	1^c^

*ED* ectodermal dysplasia, *HED* hypohidrotic ED, *EEC* ectrodactyly-ectodermal dysplasia-clefting syndrome, *AEC* ankyloblepharon-ectodermal defects-cleft lip/palate syndrome, *MGD* meibomian gland dysfunction

^a^Posterior embryotoxon, posterior pole osteomas, and bilateral peripapillary colobomas

^b^Examination under anesthesia and punctal plugs

^c^Ocular surface lesions biopsies

### Eyelid abnormalities

**Fig. 4:**
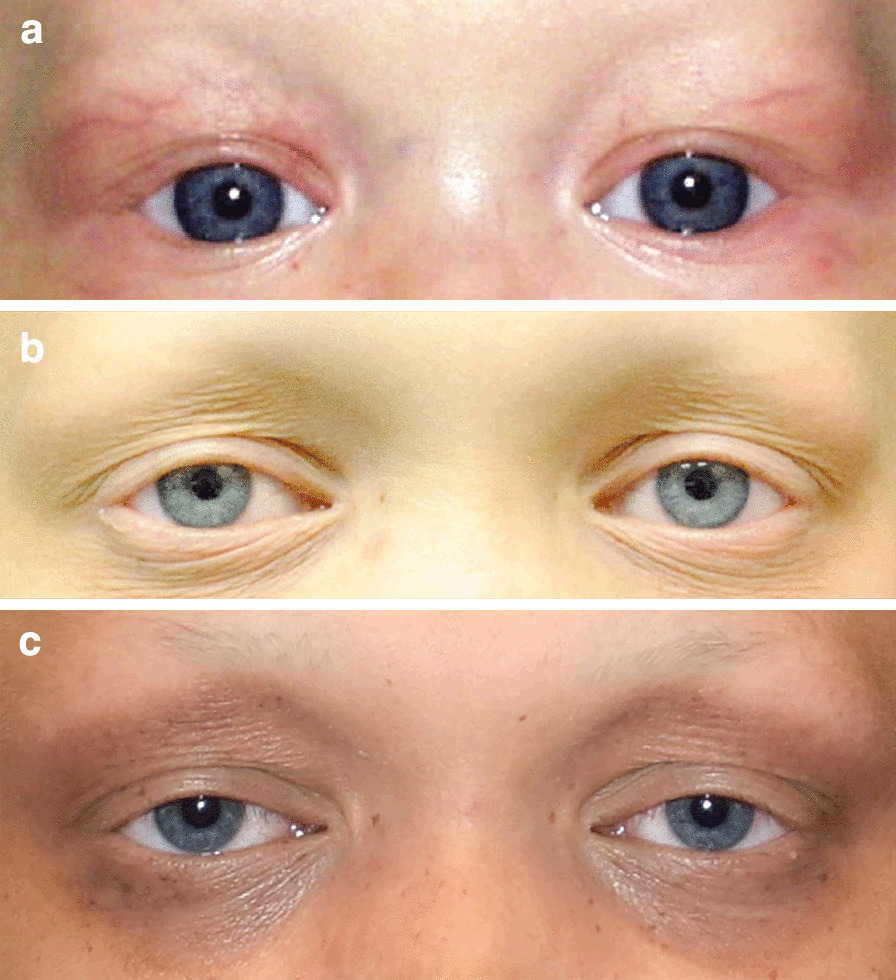
A male subject with hypohidrotic Ectodermal Dysplasia (HED) and *EDA1* gene variant, demonstrating bilateral acquired ptosis and lash ptosis. Note the typical ED presentation with light and sparse eyelashes and eyebrow hair (hypotrichosis), and progressive periocular skin changes. **a** The patient at age 5 months, with normal eyelid position. **b** Age 10, demonstrating mild bilateral ptosis, lash ptosis, and periocular skin changes. **c** Age 14, with progression of his bilateral ptosis, lash ptosis, and periocular pigmentary skin changes

Lash ptosis or ptosis were present in clinical photographs of 11 subjects (n = 11/30, 37%) at a mean age of 10 years. Four subjects had both entities, 3 had isolated lash ptosis, and 4 had isolated ptosis. Among the seven subjects with lash ptosis, genetic testing was available for three HED subjects, and were all positive for pathogenic *EDA1* gene variants. Among the subjects with primary eyelid ptosis, genetic testing was available for two subjects; one had HED with confirmed pathogenic *EDA1* gene variant (Fig. 4), and the other had AEC with confirmed *TP63* pathogenic variant.

## Discussion

Ectodermal dysplasias are genetically determined developmental defects of tissues of ectodermal origin, heterogeneous in their genetic causes and clinical phenotypes. During embryonic development, the ectoderm gives rise to the epidermis, the central and peripheral nervous system, the placodes, and neural crest cells [14]. Disruption of these tissue and cell types causes the clinical spectrum characteristic of this group of disorders. Known ocular findings in EDs include scant eyebrows and eyelashes, lacrimal gland hypoplasia, dry eyes, and blepharitis [2, 15].

Several case series focused on ocular findings in ED [6, 11–13, 16]. In the largest series, published in 2004, Kaercher [6] described a case series of 36 subjects with confirmed ED which included 30 subjects with HED, EEC syndrome (3), AEC syndrome (2), and Gorlin–Goltz syndrome (1). The author found a high percentage of dry eye (94%), eyebrows diminution (94%), lashes alterations (92%), and meibomian gland alterations (95%) detected by meibomianoscopy, and concluded that meibomian gland alterations are a reliable ocular sign of ED.

The second-largest series was published by Keklikci et al. [13] in 2014 and described 24 ED subjects at a mean age of 15.8 years (range 3–45), including HED (21 subjects) and EEC (2). Eighteen subjects (75%) suffered from ocular complaints related to the ocular surface. Eleven subjects (46%) reported irritation, tearing, epiphora, photophobia, redness, and recurrent inflammations of the lids; these were attributed to dry eye. The authors concluded that ocular complaints, particularly dry eye symptoms, may increase as age advances.

These series, as well as others, emphasized the ocular surface findings in ED, including various keratopathies, meibomian gland disorders, and dry eye [6, 12, 17, 18]. In our series, tearing secondary to lacrimal disorders including punctal agenesis was the most prevalent symptom, especially in *TP63*-related disorders (EEC, AEC, RHS), followed by photophobia secondary to dry eye, trichiasis, blepharitis, and corneal disorders.

Less than 20% of the subjects were diagnosed with dry eye, blepharitis or meibomian gland abnormalities, and no corneal dystrophies were diagnosed. These differences could be explained by the younger age of our subjects, making the history information, ocular examination and auxiliary tests more challenging. In addition, as some of these disorders develop or progress with age, these changes would be expected to be more prevalent in adulthood [13].

### Oculo-ectodermal syndrome (OES)

OES, also named Toriello Lacassie Droste syndrome, was first described by Toriello et al. [19] in 1993. About 20 cases have been reported in the literature [20, 21]. It is caused by somatic variants in the KRAS gene on chromosome [12, 21]. It is characterized by epibulbar dermoids and cutis aplasia congenital [22]. Phenotypic expression is highly variable, and various other abnormalities have occasionally been reported, including growth failure, lymphedema, cardiovascular defects, neurodevelopmental symptoms, non-ossifying fibromas of the long bones, giant cell granulomas of the jaws, arachnoid cysts in the brain, seizure disorder, hyperpigmented nevi, and rhabdomyosarcoma [20, 23].

In addition to unilateral or bilateral epibulbar dermoids, ocular anomalies such as upper eyelid skin tags, corneal opacities, hyperopia and astigmatism, strabismus, and microphthalmia can be present [21, 22, 24]. Gardner and Viljeon [25] described an affected patient with a small optic disc on the right and a large optic disc with abnormal retinal pigmentation on the left. Boppudi et al. [21] described a case with deeply set eyes and a narrow intercanthal distance, while Toriello [19] described a child with chorioretinal atrophy, prominent eyes and strabismus.

In addition to epibulbar dermoids, our subject’s ocular history included posterior embryotoxon, posterior pole osteomas, and bilateral peripapillary colobomas. These findings have not been previously described in patients with OES. Interestingly, optic nerve coloboma has been described in Encephalo‐cranio‐cutaneous lipomatosis (ECCL) [26], which supports the notion that OES may be a mild variant of ECCL [22]; furthermore, that supports an association between optic coloboma and OES.

### Eyelid malposition associated with ectodermal dysplasias

It is difficult to strictly classify the origin of individual eyelid structures into an ectodermal or mesenchymal origin [27]. Generally, the surface ectoderm gives rise to the conjunctiva, skin epithelium, hair follicles, Zeis glands, glands of Moll, and meibomian glands; the Levator aponeurosis, being of neural crest origin, is another ectodermal derivative [27]. The tarsal plate, levator muscle, orbicularis muscle, orbital septum, and tarsal muscle of Müller develop from the mesenchyme [27].

It has been advocated that gene expression in the EDs is not limited to the ectoderm and that there is a concomitant effect on developing mesenchymal structures [4, 5]. The involvement of both ectodermal and mesodermal structures in the pathogenesis of these disorders may explain the various eyelid and eyelashes abnormalities demonstrated in this series, including the high portion (37%) of eyelid ptosis and/or lash ptosis.

#### Ptosis

Ptosis has been anecdotally described in ED. Examples include Jackson and Barr (1978) [28] who described 2 sisters with ED and ptosis. Zanolli et al. [29] described ED with signs of both ectodermal and mesodermal dysplasia, associated with ptosis. Ptosis was also described in a pediatric case of Goltz syndrome, a rare ED subtype, but this was concomitant with bilateral microcorneas, microphthalmos, and iris colobomas [30]. A paper stated that ptosis is associated with AEC, but no relevant reference was recognized [2]. Salinas et al. [31] suggested ptosis as one of the manifestations of Rapp-Hodgkin syndrome.

Our findings suggest that the rate of ptosis in ED subjects, especially those with *TP63* and *EDA1* gene variants, is higher than previously demonstrated, and that such genetic associations might exist. The ptosis was noted to be acquired rather than congenital (Fig. 4). Progressive decrease in anterior lamellar tone or levator dehiscence may be the cause of eyelid malposition in our series. Actual entropion due to tarsoconjunctival contraction was not noted, however, in this series.

#### Lash ptosis

A unique observation was the high rate of lash ptosis, especially given the infrequency of this observation in the pediatric population. As previously discussed, eyelash disorders are associated with EDs, including sparse and thin lashes, pseudodistichiasis, and trichiasis [6, 32] However, lash ptosis was not previously described in ED subjects. This subtle diagnosis can be easily overlooked, especially with the HED pediatric subjects with their sparse, light eyelashes. We report a possible genetic association between the *EDA1* gene and lash ptosis.

In summary, EDs are associated with various ocular and ocular adnexal abnormalities. In this series, several new findings were observed, including increased risk of amblyopia in most subtypes, as well as eyelid ptosis and lash ptosis in subtypes with *TP63* or *EDA1* gene variants. In addition, trichiasis may be more common in AEC than previously described, and posterior pole osteomas and peripapillary colobomas may be found with OES. These various findings mandate early ophthalmic evaluation in this unique group of children.

## Data Availability

Data available on request.
